# Future changes in the precipitation regime over the Arabian Peninsula with special emphasis on UAE: insights from NEX-GDDP CMIP6 model simulations

**DOI:** 10.1038/s41598-023-49910-8

**Published:** 2024-01-02

**Authors:** K. Koteswara Rao, Abdulla Al Mandous, Mohamed Al Ebri, Noora Al Hameli, Mohamed Rakib, Shamsa Al Kaabi

**Affiliations:** 1 National Center of Meteorology (NCM), P.O. Box 4815, Abu Dhabi, United Arab Emirates; 2 World Meteorological Organization (WMO), P.O. Box 2300, Geneva, Switzerland

**Keywords:** Atmospheric science, Climate change, Climate sciences

## Abstract

Global warming can profoundly influence the mean climate over the Arabian Peninsula, which may significantly influence both natural and human systems. The present study aims to investigate the changes in the precipitation regime in response to climate change over the Arabian Peninsula, with special emphasis on the United Arab Emirates (UAE). This work is performed using a sub-set of high-resolution NASA Earth Exchange Global Daily Downscaled Projections (NEX-GDDP) data derived from Coupled Model Intercomparison Project Phase 6 (CMIP6) Global Climate Models under three different Shared Socioeconomic Pathway (SSP) scenarios (SSP1-2.6, SSP2-4.5, and SSP5-8.5). The changes are analyzed in three phases such as 2021–2050 (near future), 2051–2080 (mid future) and 2080–2100 (far future), with the period of 1985–2014 as the baseline. This study represents the first attempt to utilize data from NEX-GDDP models to project the regional patterns of precipitation regime across the Arabian Peninsula. Results suggest that the annual precipitation is expected to increase over most of the UAE by up to 30%, particularly intense from the mid-future onwards in all scenarios. Specifically, the spatiotemporal distribution of precipitation extremes such as intensity, 1-day highest precipitation, and precipitation exceeding 10 mm days are increasing; in contrast, the consecutive dry days may decrease towards the end of the century. The results show that the changes in extreme precipitation under a warming scenario relative to the historical period indicate progressive wetting across UAE, accompanied by increased heavy precipitation events and reduced dry spell events, particularly under the high emission scenarios. A high-resolution dataset is essential for a better understanding of changes in precipitation patterns, especially in regions where more detailed information is needed on a local scale to achieve water, food security, and environmental sustainability to formulate effective adaptation strategies for mitigating the potential risks and consequences associated with variations in wet and dry conditions.

## Introduction

The Earth's changing climate significantly influences life, resources, and the environment. A changing climate can result in unprecedented extreme weather and climate events^1,2^. According to the recent IPCC report^3^, the world's average temperature rise is likely to reach or exceed 1.5 °C between 2030 and 2050. In the past few decades, the rising levels of anthropogenic greenhouse gases (GHGs) have been attributed to the unusual warming of the Earth. This has resulted in more frequent extreme temperatures and intense precipitation events, which tend to last longer. These changes have significantly affected various aspects of our living and working environments^4^. It is also expected that global warming could manifest differently on a regional scale, where the expected future changes might differ from place to place around the world; Trenberth^5^ has observed the changes in mean annual precipitation in different regions of the world, particularly the tropics and subtropics of the Northern Hemisphere (NH) showed decreasing tendencies. However, precipitation projections across different regions exhibit large spatial variability and seasonal changes^6–8^. In recent decades, various regions across the globe have experienced a notable increase in the occurrence of life-threatening rain events, including both floods and droughts^4^. An increase in human-induced greenhouse gases has been linked with a widespread increase in the frequency and intensity of daily precipitation extremes across the globe^9^.

The Arabian Peninsula, one of the regions exposed to climate change, has experienced a growing number of severe extreme events, such as heavy floods, prolonged droughts, and drying lakes, in recent decades^10^. These climate-related issues have exacerbated the region's environmental, agricultural, and public health issues^11,12^. Water stress is currently being observed in the Arabian Peninsula, and as a result, desalinated water from the Arabian Gulf is being extensively utilized to meet the water needs of the surrounding cities. However, this increased reliance on desalination has led to additional environmental stress on the water body itself^13,14^. Moreover, the precipitation patterns over the Arabian Peninsula are influenced by the considerable spatial variability in precipitation. This variability is attributed to diverse climatic influences such as the Mediterranean climate, Indian monsoon, and orographic variations^15^. Despite its importance, the precipitation mechanisms over this region have yet to be explored in detail in previous studies, primarily due to a scarcity of observations^16^. The likely expansion of desert regions in response to a warming climate^17^, the Arabian Peninsula is recognized as a region that is particularly sensitive to the impacts of climate change^18,19^; it is crucial to have a good understanding of the local conditions to predict the future climate better. In this regard, Evans^20,21^ emphasizes the importance of comprehending the precipitation variability over the Arabian Peninsula to make reliable assessments of water stress in a warming climate. This understanding becomes particularly critical as the spatial distribution of changes in precipitation patterns in response to climate change.

In the past few decades, the UAE and adjoining regions have rapidly developed in terms of industrialization, urbanization, and living standards with modern amenities^22^. The changing climatic conditions may affect various aspects of the UAE’s critical infrastructures, energy, and agriculture sectors. Therefore, estimating possible future changes in weather and climate is essential for fostering climate-resilient development in the UAE and adjacent regions. It enables proactive decision-making and facilitates the implementation of transformative measures that build resilience, mitigate risks, and enhance sustainability in the face of a changing climate. Understanding these impacts is vital for building adaptive capacity and enhancing climate resilience^23,24^. Despite the significance of climate change concerns, more studies should focus on this region. Given the vulnerability of countries like the UAE to climate change impacts, it becomes crucial to understand the potential future changes, especially regarding extreme weather events. This in particularly important for a country like the UAE, where various outdoor activities occur in sectors such as onshore/offshore energy, extensive construction, and agriculture. Hence, understanding the spatial and temporal dynamics of precipitation changes concerning global warming becomes crucial. By studying these characteristics, we can better comprehend how precipitation patterns may evolve in response to climate change.

The interaction of land, ocean, and atmosphere in response to past, present, and future global warming is needed to understand the impact of climate change at spatial–temporal scales. The General Circulation Models (GCMs) are widely acknowledged as the primary instruments used to comprehend historical climate patterns project future climate simulations and their impacts^25^. The most recent CMIP6 dataset provides a collection of advanced climate model experiments that offer valuable insights into various climate responses and mechanisms. However, applying GCMs at the regional level is a challenging task due to their limited resolution and inability to capture sub-grid scale processes accurately^26^. The GCMs are known to exhibit notable biases in seasonal averages, and these biases vary in space and across different models^27–29^. In addition, it cannot adequately capture extreme events like intensity, frequency, and spatial distribution^9,30^. In response to the demand for more precise regional climate projections, various dynamical and statistical downscaling techniques have emerged, offering higher-resolution data to enhance reliability^24,31^. Numerous researchers^32–35^ have emphasized the importance of employing downscaling and bias correction techniques to obtain high-resolution datasets for generating accurate climate change projections at the regional scale. These two methods are commonly utilized to enhance the accuracy of regional climate projections.

The National Aeronautics and Space Administration (NASA), through the NEX-GDDP project, has recently created advanced datasets incorporating state-of-the-art bias correction and statistical downscaling techniques^31^. These datasets have been derived from CMIP6 model outputs and offer high-resolution, reliable information under three distinct Shared Socioeconomic Pathways (SSPs). Jain et al.^36^ assessed the NEX-GDDP dataset with the CMIP5 dataset specifically for the Indian subcontinent. They discovered significant improvements in the NEX-GDDP dataset, highlighting its potential for future projections and impact studies at the regional level. In addition, the authors emphasized the importance of high-resolution data in obtaining reliable climate information at the local and regional scales, which is decisive for developing effective adaptation strategies^37^. The advantage of high-resolution regional climate simulations lies in their ability to evaluate climate change impacts on processes sensitive to fine-scale climate gradients and the influence of local topography on climate conditions^31^.

The present work is the first attempt to use NEX-GDDP models data to address projections of future mean and extreme precipitation changes in the UAE. These projections have critical societal implications for coping with future climate change in the UAE and adjoining areas. This understanding will aid policymakers in quantifying the potential impacts of extreme events and enable them to develop suitable adaptation strategies. These future scenarios can provide valuable insights for societal stakeholders and decision-makers with science-based climate information to adequately adapt to expected impacts and build tomorrow's climate-resilient societies.

The rest of the paper is structured as follows: “Data and Methods” Section provides an overview of the data and methods used in this study. In “Results and discussion” Section, we analyze the historical and projected changes in precipitation patterns over the UAE. Lastly, “Summary and conclusions” Section presents the summary and conclusions of the study.

## Data and methods

The United Arab Emirates (UAE), which is located in the Arabian Peninsula between latitudes 22.35 °N and 26.50 °N and longitudes between 51.35 °E and 57.10 °E. The UAE has an arid desert climate (Koppen climate classification, *Bwh*)^38^, characterized by a mild winter and a hot summer. Winter is cooler, with occasional rainfall. Spring and autumn seasons are warm, predominantly dry, and pleasant in terms of weather conditions. Summer rainfalls tend to be lower, particularly along the coastal and inland areas, away from the mountains. A hot, dust-laden wind blows from March to August in the spring and summer. These winds can be very strong and cause sandstorms throughout the year. The country’s annual mean rainfall is under 100 mm per year, and on most occasions, the mountainous areas receive a higher amount of rainfall^39–43^. In addition, thunderstorm activity contributes most of the precipitation in this region due to the passage of western upper air troughs during wintertime^44,45^. In a warming world, mesoscale convective systems occurring over the southern Arabian Peninsula are anticipated to have an even greater impact, particularly in terms of extreme precipitation events^43^.

A suite of thirty statistically downscaled, bias-corrected, and high-resolution simulations of precipitation data (0.25° × 0.25°) from the NEX-GDDP project has been used to assess future climate. These datasets are derived from the new generation of state-of-the-art GCM simulations made under Coupled Model Intercomparison Project Phase 6 (CMIP6). The Bias-Correction Spatial Disaggregation (BCSD) method^25,31,46^ is adopted to generate these datasets. The NEX-GDDP project focuses on enhancing the CMIP6 simulations by extending their applicability from large-scale projections to regional and local scales^31^. The information on these NEX- GDDP model datasets is provided in Table 1.

**Table 1:** List of modeling groups and models used in this study (source: Thrasher et al. 2022).

Modeling Center (or Group)	Institute ID	Model name
Commonwealth Scientific and Industrial Research Organization (Australia)	CSIRO-BOM	ACCESS-CM2 ACCESS-ESM1-5
Beijing Climate Center (China)	BCC	BCC-CSM2-MR
Canadian Centre for Climate Modelling and Analysis (Canada)	CCCMA	CanESM5
National Center for Atmospheric Research (USA)	NCAR	CCSM2 CESM2-WACCM
ondazione Centro Euro-Mediterraneo sui Cambiamenti Climatici (Italy)	CMCC	CMCC-CM2-SR5 CMCC-ESM2
Centre National de Recherches Météorologiques (France)	CNRM-CERFACS	CNRM-CM6-1 CNRM-ESM2-1
EC-Earth consortium, Rossby Center, Swedish Meteorological and Hydrological Institute/SMHI (Sweden)	EC-EARTH	EC-Earth3 EC-Earth3-Veg-LR
Chinese Academy of Sciences (China)	FGOALS	FGOALS-g3
NOAA Geophysical Fluid Dynamics Laboratory (USA)	NOAA GFDL	GFDL-CM4 (gr1) GFDL-CM4 (gr2) GFDL-ESM4
Goddard Institute for Space Studies, National Aeronautics and Space Administration (USA)	GISS	GISS-E2-1-G
Met Office, Hadley Centre (UK)	HadGEM	HadGEM3-GC31-LL HadGEM3-GC31-MM
Indian Institute of Tropical Meteorology (India)	IITM	IITM-ESM
Institute for Numerical Mathematics (Russia)	INM	INM-CM4-8 INM-CM5-0
Institut Pierre-Simon Laplace (France)	IPSL	IPSL-CM6A-LR
National Institute of Meteorological Sciences (NIMS) and Korea Meteorological Administration (KMA) (South Korea)	KACE	KACE-1–0-G
Korea Institute of Ocean Science and Technology (South Korea)	KIOST	KIOST-ESM
Japan Agency for Marine-Earth Science and Technology (Japan)	MIROC	MIROC-ES2L MIROC6
Max Planck Institute for Meteorology (Germany)	MPI-M	MPI-ESM1-2-HR MPI-ESM1-2-LR
Meteorological Research Institute (Japan)	MRI	MRI-ESM2-0
Nanjing University of Information Science and Technology	NESM	NESM3
Norwegian Climate Centre (Norway)	NCC	NorESM2-LM NorESM2-MM
Research Center for Environmental Changes, Academia Sinica (Taiwan)	TaiESM	TaiESM1
Met Office, Hadley Centre (UK)	UKESM	UKESM1-0-LL

The historical simulations from the period from 1950 through 2014 and the future projections are utilized for the period 2015–2100 under the SSP1-2.6 (low emission), SSP2-4.5 (medium emission), and SSP5-8.5 (high emission) scenarios^47^. During the historical simulations, the climate models were subjected to anthropogenic influences, including greenhouse gases, anthropogenic aerosols, and external natural factors such as solar and volcanic activities. In contrast, for climate projections beyond the year 2014, different Shared Socioeconomic Pathways (SSPs) drove the simulations^48^. The latest Shared Socioeconomic Pathways (SSPs) encompassed explicit modifications in indicators of societal development, such as population and economy. These pathways were designed to achieve a stable carbon dioxide concentration and respond to radiative forcing over the course of the next century.

For assessing the present and future climate, the twenty-first century is considered into four different periods—the historical period (1985–2014), the near future (NF; 2021–2050), the mid-future (MF: 2051–2080), and the far future period (FF; 2081–2100). Given that the present study aims to present spatially/temporally uniform information on observed Arabian precipitation extremes, an appropriate gridded dataset is required. The daily precipitation data from Climate Prediction Center (CPC) developed by the National Oceanic and Atmospheric Administration (NOAA) at 0.25° × 0.25° spatial resolution for 1979 to 2022 are used to understand the observed variability. For developing this dataset, CPC has used the optimal interpolation of quality-controlled gauge records of the Global Telecommunication System (GTS) network^49,50^. The CPC is also one of the available gauge-based, gridded daily dataset for the study area. The CPC dataset has gained widespread usage for observational studies worldwide. These observations provide reliable data subject to the quality control process, which has been developed after following quality control of the station data, and more details of the data development are available on the CPC website (https://psl.noaa.gov/data/gridded/).

The model simulations and observational data have been standardized to a uniform grid resolution to ensure consistency and comparability. This standardization allows for the computation of the Multi-Model Mean (MMM), which effectively reduces random errors inherent in individual models and dampens the influence of internal variability^51^. This paper focuses on the MMM to ensure robustness in assessing and diagnosing precipitation changes at both spatial and temporal scales in the current and future climate. By relying on the collective information provided by multiple models, a more precise understanding of precipitation patterns can be obtained, enhancing the reliability of the findings presented in the study. Recently, numerous climate indicators have been developed to examine climate-induced extremes. Most climatological studies adhere to the guidelines set by the Expert Team on Climate Change Detection and Indices (ETCCDI). The ETCCDI has established a comprehensive set of climate change indices specifically designed to assist in analyzing and investigating extreme events^52^. In this paper, the analysis focuses on a subset of four key precipitation indices^53,54^ (see Table 2). Considering a range of scenarios, this analysis provides a comprehensive overview of the anticipated changes in precipitation extremes. The study examines various projected future conditions to understand how extreme precipitation events may evolve under different climate scenarios. These FOUR key indices are based on the daily precipitation amounts and their details are given in Table 2.

**Table 2:** List of precipitation indices analyzed in the study.

Indices	Description	units
SDII	The daily precipitation amount averaged over all wet days in a year	mm/day
RX1DAY	Annual maximum 1-day precipitation amount	mm
PD10MM	Annual count of days with precipitation greater than 10 mm	Days
CDD	Maximum number of consecutive days with precipitation < 1 mm	Days

In this study, all the indices mentioned have been calculated annually for each climate model, subsequently, computed the MMM for historical and future climate periods. The annual trends in these precipitation extremes are calculated using the Sen Slope^55^. The significance of trends is evaluated using the non-parametric Mann–Kendall (MK) test^56,57^. This statistical test is employed to assess the significance of trends in the data at a predetermined level of statistical significance, typically set at *p* < 0.05.

It is suitable for precipitation data, as it does not assume the underlying distribution. In addition, we used statistical metrics to evaluate the models' performance and associated diagnostic indices for 30 years from 1985 to 2014 (Table 2). The CPC and individual CMIP6 model data over UAE are used for model evaluation of precipitation and diagnostic indices, respectively.

The interannual variability skill score^58–60^ between the models simulations relative to the observations calculated for the historical period. The interannual standard deviation assesses the variability. The IVS is calculated as

1 $$IVS = \left( {\frac{{STD_{m} }}{{STD_{o} }} - \frac{{STD_{o} }}{{STD_{m} }}} \right)^{2}$$

where STD_m_ and STD_o_ indicate the interannual standard deviation of the model simulation and observation. An IVS score closer to zero indicates that the model chosen is performing closer to the observations in terms of interannual variations. IVS, a symmetric variability ability statistic, measures how closely interannual variation in simulations and observations resembles each other.

In addition, the relative error was calculated between the precipitation outputs of each CMIP6 models (including the multi-model mean) and the CPC dataset.

2 $$RE = \frac{{\overline{X}_{m} - \overline{X}_{o} }}{{\overline{X}_{o} }} \times 100\left( \% \right)$$

where x̅_m_ and x̅_o_ indicate the mean of model and observation.

The values of IVS and RE closer to zero indicate that the model simulates similarly to the observations.

## Results and discussion

The results are primarily analyzed and presented in three main categories, i.e., observed variability, model validation for the historical period, and future climate projections. The model simulations are validated against the corresponding observations on both climatological (long-term average) and interannual (year-to-year variability) time scales. Further, the future changes in the precipitation regime relative to the historical periods have been analyzed in detail.

### Observed precipitation variability

**Figure 1:**
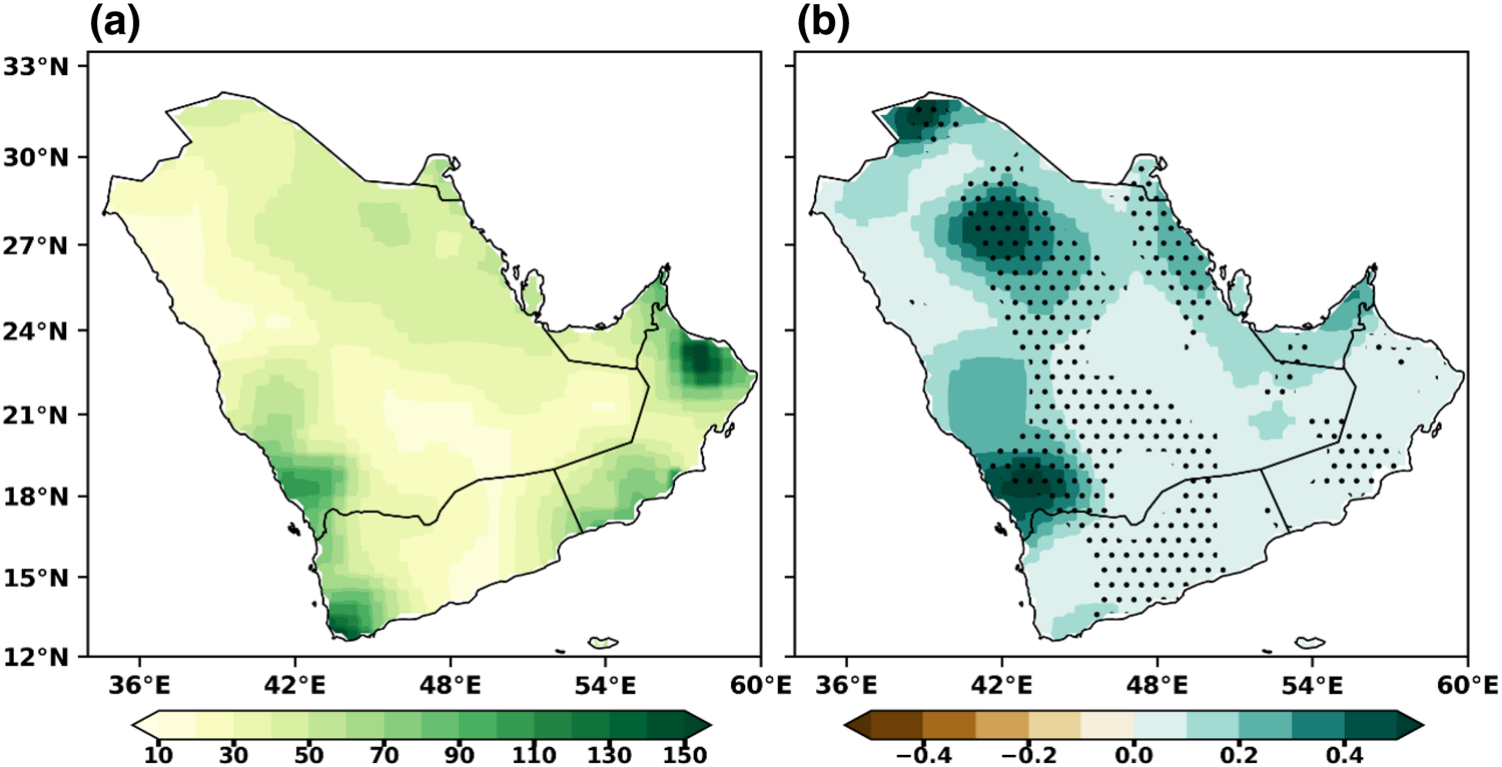
The (**a**) mean climatological pattern (mm) and (**b**) linear trend in annual rainfall (mm/decade) based on 1979–2022. The stippling (dotted) regions represent the trend is statistically significant at 0.05 level.

**Figure 2:**
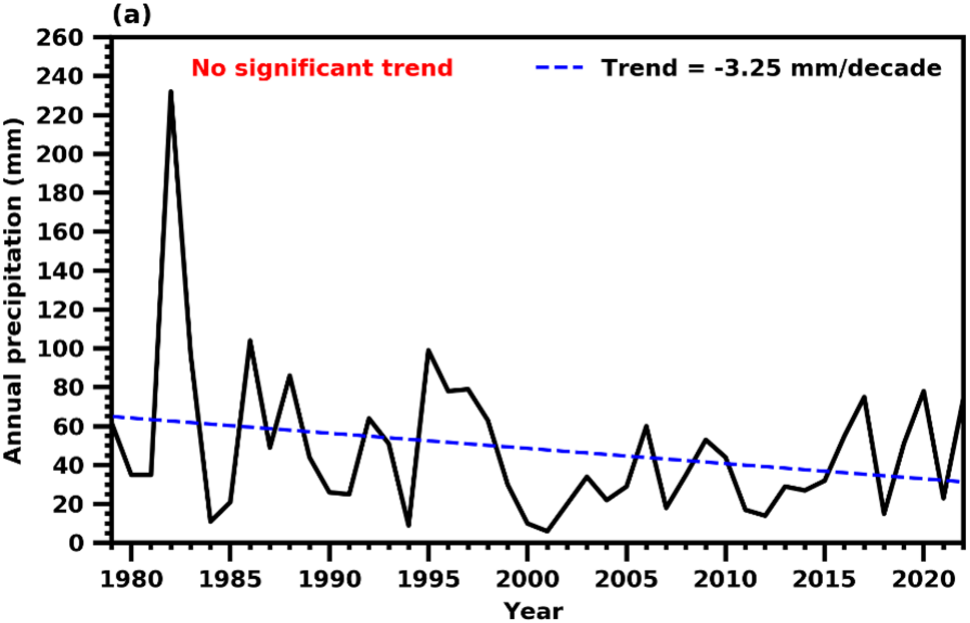
Inter-annual variability of area averaged UAE annual total precipitation. The blue color depicts linear trend.

The annual mean precipitation (during 1979–2022) ranges from 40 mm in the desert areas to 130 mm over the coastal and mountain regions of the Arabian Peninsula, while most of the interior peninsula received up to 50 mm/year (Fig. 1a). A significant decreasing trend in annual mean precipitation was noticed in most of the peninsular areas (Fig. 1b). The precipitation climatology clearly shows excellent spatial and temporal diversity in rainfall (Fig. 1a), and topography strongly governs rainfall climatology due to its arid environment nature^61^. A detailed description of the precipitation climatology in the Arabian Peninsula can be found in^18,62^. The climatological annual mean rainfall over UAE is under 110 mm/year; on most occasions, the mountainous areas in the northeastern parts receive more rain (Fig. 1a). The area average annual precipitation time series over the UAE does not show any significant trend in recent periods. However, there was a slight decrement in the precipitation with 4.2 mm /decade over the entire UAE (Fig. 2), and the country faced a plunging shift in annual rainfall after 1999 onwards. Ouarda et al.^44^ studied the inconsistency of rainfall over UAE and found that the annual precipitation series showed decreasing trends, although often insignificant.

**Figure 3:**
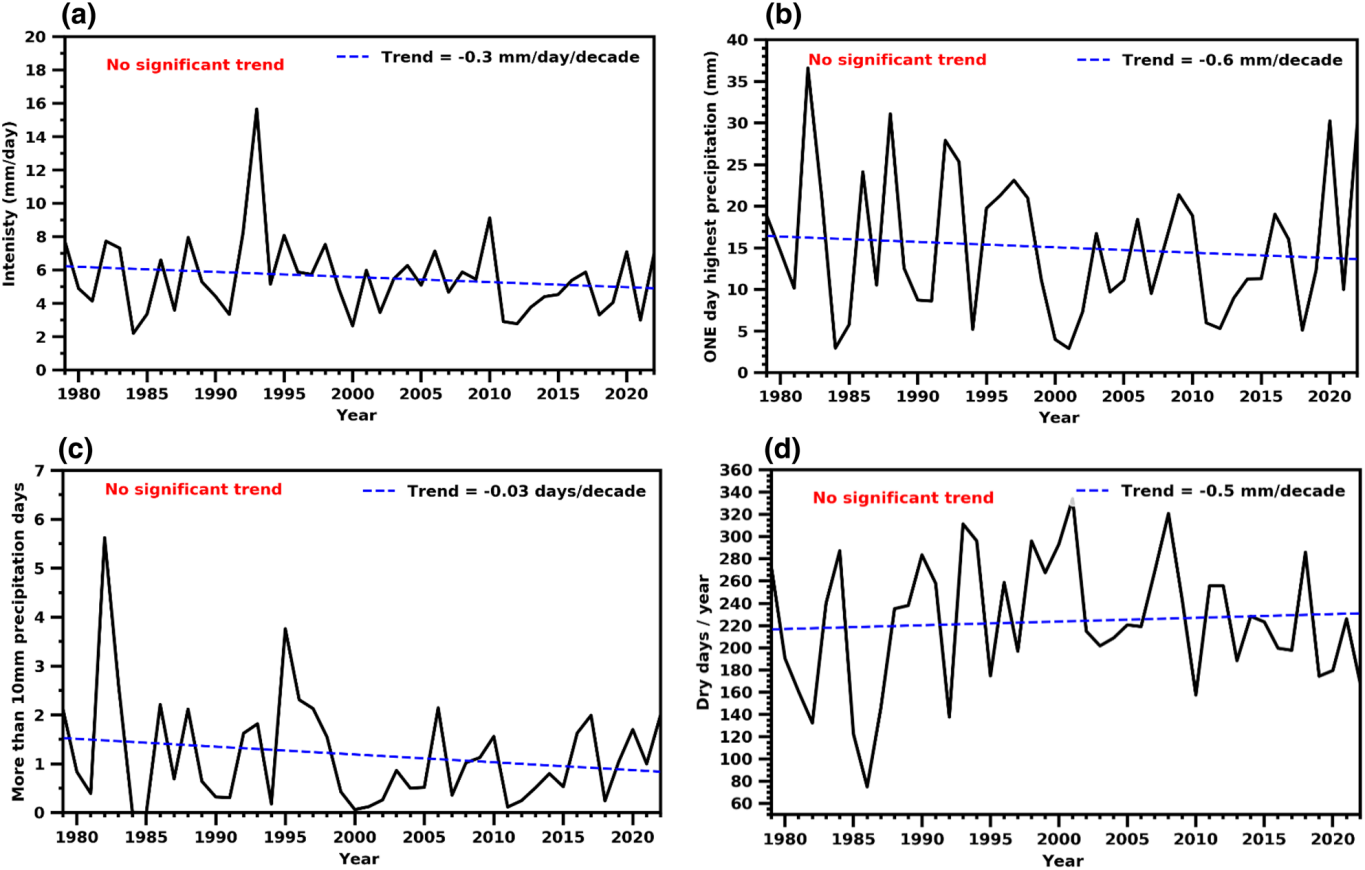
Inter-annual variability of SDII, RX1DAY, PD10 and CDD over UAE. The blue line indicates linear trend over 1979–2022.

Further, the study highlights the presence of high spatial and temporal variability in precipitation patterns, along with its standard deviation occasionally exceeding the mean annual precipitation amount. The knowledge of precipitation extremes becomes substantial to understand precipitation regimes in the country adequately. Chowdhury and Al-Zahrani^63^ mentioned that extreme daily precipitation events contribute to about 20–70% of the total precipitation in the UAE with a shorter duration. Here, we present four extreme precipitation indices (viz. intensity, one-day highest rainfall, more than 10 mm precipitation days, and dry days) over UAE using CPC gridded precipitation data sets for 1979–2022. The four precipitation indices do not show any significant trend over 44 years (1979–2022). However, slight increasing/decreasing tendencies were noticed (Fig. 3).

### Historical simulations and future climate change projections

#### Historical model simulations

**Figure 4:**
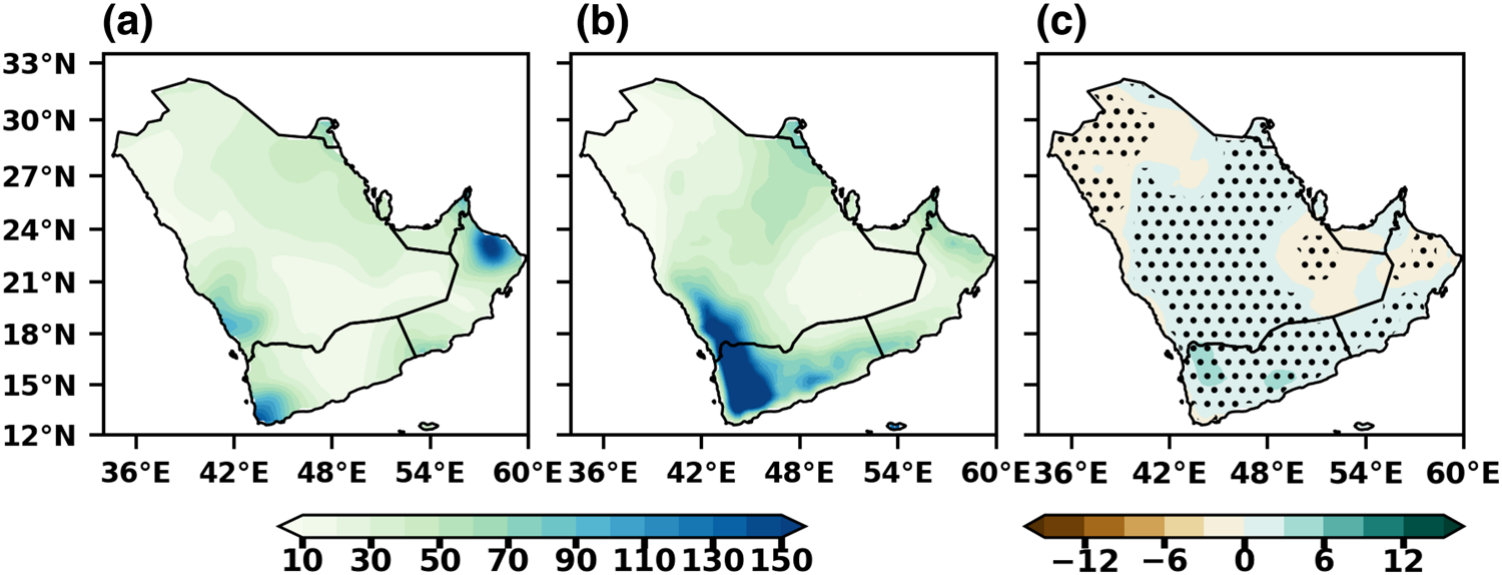
Spatial distribution of annual mean precipitation over Arabian Peninsula during 1985–2014 (**a**) CPC (**b**) CMIP6 MMM and (**c**) BIAS. The stippling (dotted) regions represent the biases are statistically significant at 0.05 level.

The MMM annual mean precipitation climatology distribution is assessed with the CPC data for the historical period (1985–2014), as shown in Fig. 4. The left (Fig. 4a) and middle (Fig. 4b) represent the mean precipitation based on CPC and MMM, while the right (Fig. 4c) shows the bias between them. The MMM shows a similar spatial pattern as in the observed precipitation and reasonably reproduces the present climate over the Arabian Peninsula, except over the southwest peninsula (Fig. 4b). Both the CPC and MMM capture the mean precipitation pattern and bias is found to be stronger in the high mean precipitation regions (Fig. 4c). The simulated annual mean precipitation is around 10–120 mm/year over the coastal and mountain regions over the peninsula. A bipolar bias was noticed in the MMM, i.e., dry bias over the central and northern parts, whereas wet bias over the southern parts of the peninsula. Slight dry biases of around 5% have been noticed over the central and northern parts of the peninsula, which indicates that the MMM underestimated the precipitation in those areas; however, the complex nature of this region and these biases in the precipitation patterns can be attributed, in part, to the uncertainties present in the observation dataset. The statistically downscaled, bias-corrected datasets exhibit a notable reduction in biases compared to the original CMIP6 datasets. The bias values in these downscaled datasets are generally within the range of ± 10% for most locations. The results show that the MMM can provide a more reliable estimation of mean precipitation than individual models while comparing with the observations. Since the MMM simulates the annual rainfall well, we can have considerable confidence in the projections. The stippling (dotted) regions represent the biases are statistically significant at 0.05 level. Almazroui et al.^7^ also found slight variation between the observed datasets and the CMIP6 models, and overall precipitation distributions remain almost the same over Arabian Peninsula. Previous studies by Zittis et al.^64^ and Bucchignani et al.^15^ mentioned dry bias in precipitation over North Africa and the Arabian Peninsula with RCM simulations. In contrast, Voldoire et al.^65^ found slight biases in most cases, suggesting that the downscaled datasets provide a more accurate representation of the atmospheric circulation patterns in the retrospective simulations, enhancing our understanding of the climate system in the Arabian Peninsula.

**Figure 5:**
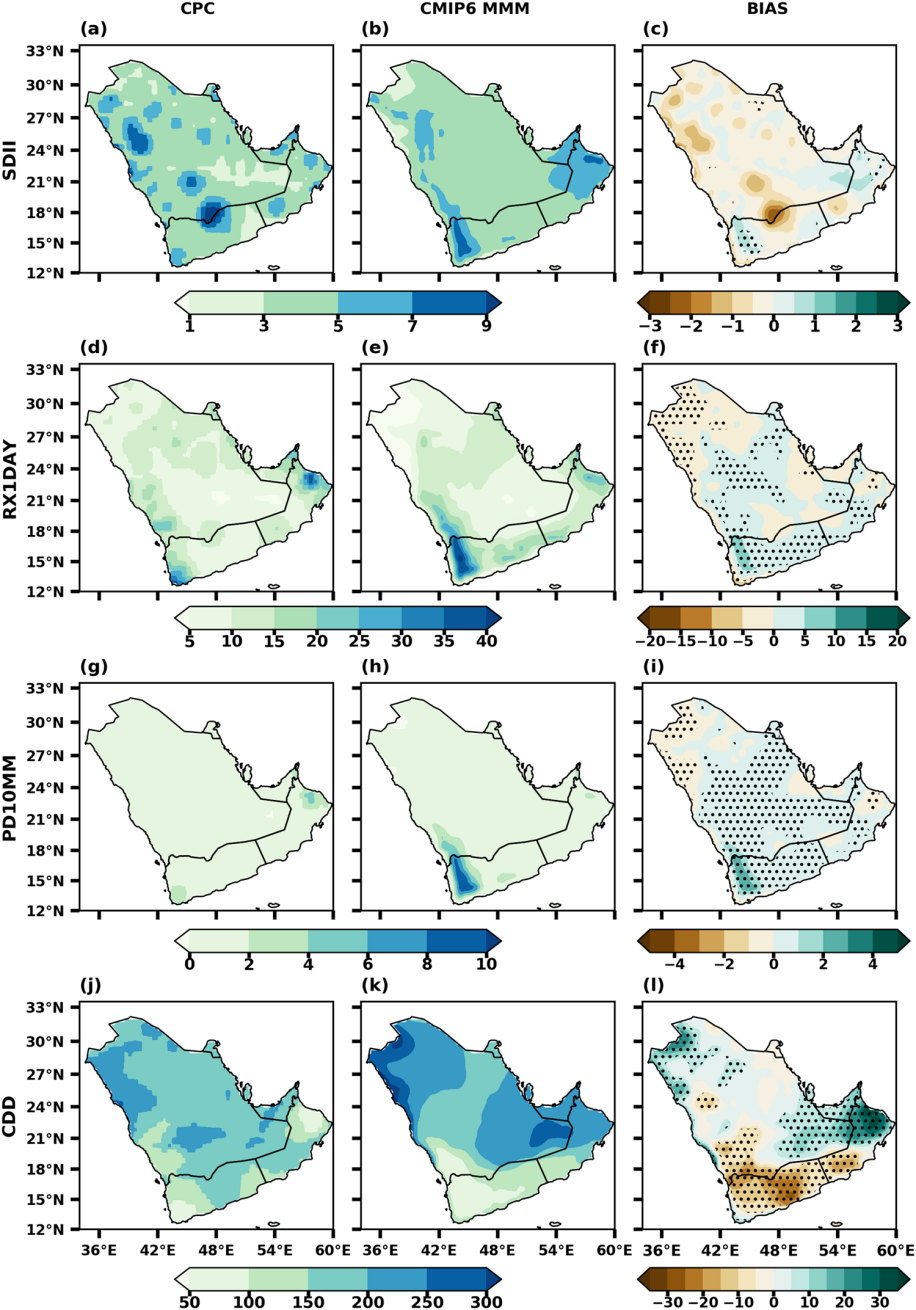
Spatial distribution of extreme precipitation indices over Arabian Peninsula CPC (1st column), CMIP6 MMM (2nd column), and BIAS (3rd column) during 1985–2014. The stippling (dotted) regions represent the statistically significant biases at 0.05 level.

The spatial patterns of precipitation indices such as SDII, RX1DAY, PD10MM, and CDD from the MMM and CPC, along with their biases for the baseline period (1985–2014), are analyzed (Fig. 5). Most precipitation extreme indices (SDII, RX1DAY, and PD10MM) show similar spatial variations over the peninsula except for the CDD, which showed a higher variability. The intensity of precipitation (SDII) is well simulated by the MMM over the observed CPC data. Maximum intensity is seen over mountain regions of UAE and Oman and similarly over southern areas of the peninsula (Fig. 5b). The MMM shows bipolar bias, i.e., wet bias over the south and NE regions and dry bias over central through northern parts of the peninsula. The MMM of RX1DAY has a similar pattern with observation (Figs. 5d,e) with a slight positive bias. The RX1DAY is more simulated by 30–50 mm over coastal and mountain regions, especially the southern and southeastern parts. The MMM are reasonably well simulated one-day highest precipitation amounts (Fig. 5e), and these are exhibiting similar features like precipitation intensity. The MMM simulates heavy precipitation days (PD10mm) over some locations in the southern peninsula with a range of 6–14 days. The remaining areas are simulated with less than six days (Fig. 5h). The MMM captures the distribution of PD10MM during the period and resembles the observed data with a slight positive bias. The spatial pattern of the number of consecutive dry days (CDD) is well simulated by the MMM (Fig. 5d). The maximum number of dry days over the central and northern parts of the peninsula gradually decreases from north to south. A slight underestimation in CDD occurred in the south and southeast, whereas, in the remaining parts of the peninsula, it could have been more overestimated relatively with observations. These findings gave confidence to suggest that the NEX-GDDP dataset can accurately reproduce the spatial patterns of precipitation extremes across the Arabian Peninsula.

The observed precipitation indices show a greater spatial extent over the region, which infers the underestimation of these indices by MMM and these patterns are similar to Al Sarmi et al.^66^. However, the MMM reasonably well captured the mean climatological patterns of these precipitation indices. The findings reveal a notable shift in the occurrence and intensity of precipitation extremes across numerous areas within this region. The results indicate a significant change in the frequency and intensity of extreme precipitation events, highlighting a departure from previous patterns. The northern regions of the UAE have encountered instances of extreme precipitation, primarily attributed to the interplay of deep convection induced by tropical and extratropical influences, as well as the region's topography. These conditions have often led to heavy rainfall episodes, which have resulted in significant flooding events with severe impacts on human life and the economy^67^.

However, there is an increasing trend over the southern Arabian Peninsula in the winter^42,68^ and summer seasons^69^. Al Mazroui^67^ results over the same region show a slight in-phase and out-of-phase in the extreme precipitation indices. In the MENA region, the CMIP5 GCMs and CORDEX RCMs ensembles overestimated the frequency of extreme precipitation events compared to observations^70,71^. Zittis et al.^72,73^ identified significant differences between observational and reanalysis products in the case of exceptional extreme rainfall events for the MENA region. Overall, the pattern of simulated precipitation closely follows the observed pattern obtained from CPC data over many areas in the peninsula.

The above analysis shows that the model performance generally varies from model to model from one extreme index to another (Figs. 4 and 5). Thus, describing the model skill in simulating precipitation indices needs further attention. To understand the model evaluation, two additional metrics, i.e., Interannual Variability Skill (IVS) and Relative Error (RE), are used to the model performance on precipitation over UAE and associated diagnostic indices for 30 years from 1985 to 2014. First, the IVS^74^ used to verify whether the models could simulate the interannual variability in the observations (Eq. 1). Furthermore, relative error (RE) was used to check the mean error for each model (Eq. 2). The performance in simulating the temporal variation is also a crucial factor in measuring the capability of models.

**Figure 6:**
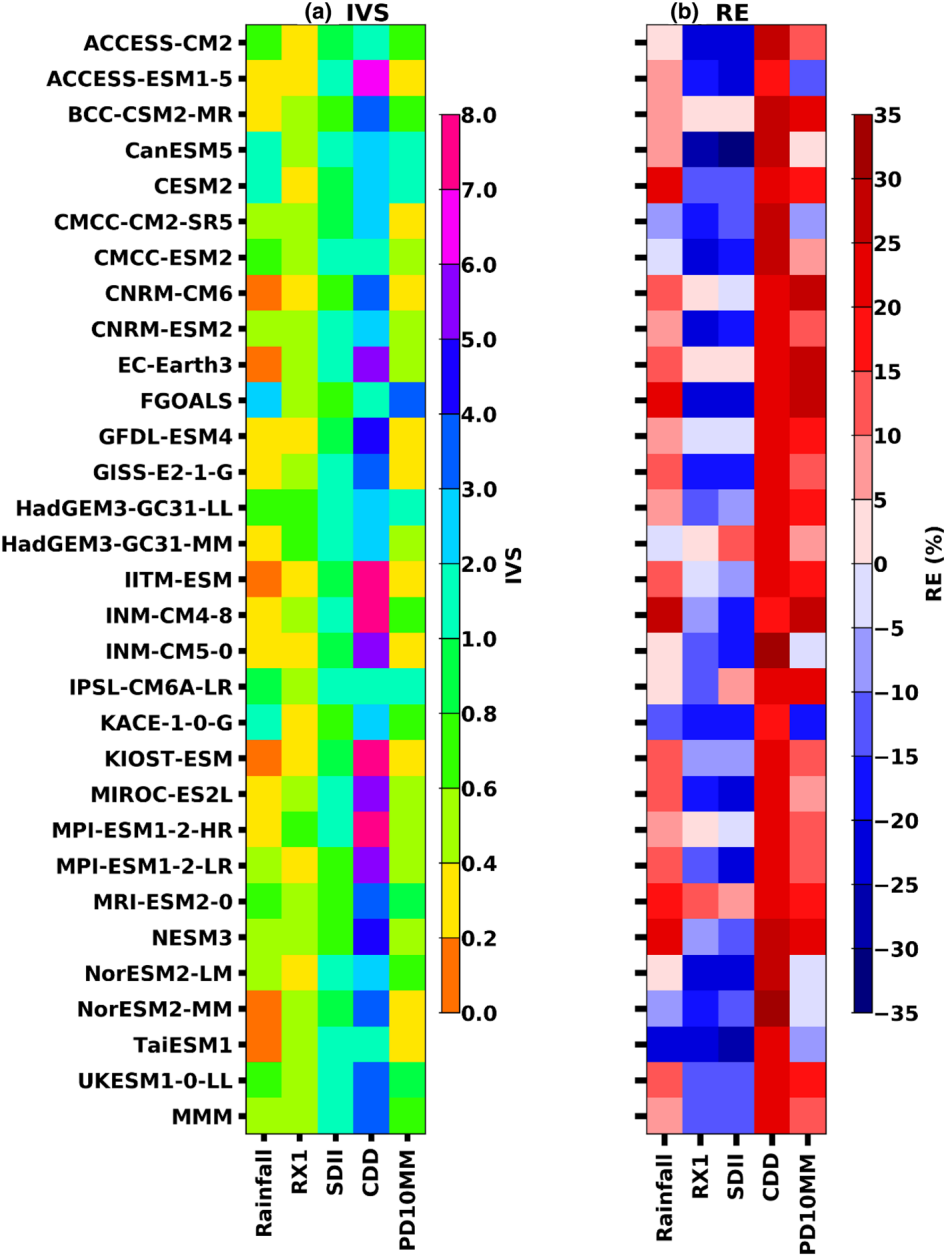
A portrait diagrams for Interannual variability Score (IVS) and Relative Error (RE) showing the performance of each single model for precipitation extreme indices area averaged over UAE during the period 1985–2014.

Figure 6a represents the IVS values of the individual model simulations and their MMM for annual precipitation and indices over the UAE. The IVS values are first calculated at each grid and then averaged. The findings suggest a substantial variation among models in their capacity to simulate precipitation extremes. In Fig. 6a, the IVS values are well below 2.0 in most individual models for all the indices except for CDD, which shows a value of around 7.0. It indicates inconsistencies among the models in CDD, whereas their MMM reproduces reasonably well with interannual variability. Low Index of Agreement (IVS) values indicate a reasonable reproduction of the observed interannual variability, which is apparent in the MMM in all the precipitation indices. Furthermore, the model performances in reproducing the interannual variability of annual precipitation exhibit better results in the Multi-Model Mean (MMM) compared to individual models.

Further, we evaluated the Relative Error (RE) of precipitation indices between the individual models and observations for the historical period over UAE (Fig. 6b). Please note that positive and negative values of the Relative Error (RE) indicate the relative extent of overestimation and underestimation, respectively. A zero value represents a perfect match between the model simulation and the observed data. When considering the Multi-Model Mean (MMM), it is observed that the differences between individual models are insignificant across various precipitation variables and associated indices. Most individual models overestimate/underestimate the precipitation indices, but the MMM shows 15% of RE. The annual precipitation simulates observed features relatively well with < 5% of RE, while RX1 and SDII are underestimated in most models; as a result, MMM is also underestimated. For example, the CDD performs relatively higher in most models than the MMM. RE and IVS can explain the resemblance between the observations and model simulations.

Furthermore, a correlation exists between a model's relative skill in simulating the mean climate and its performance in capturing the interannual variability and the relative errors. In summary, the Multi-Model Mean (MMM) results outperform individual models across all indices, indicating that the MMM provides a more reliable representation of future projections by significantly reducing structural model uncertainties. The results also demonstrate that the NEX-GDDP models exhibit a closer alignment with observations and display reduced relative errors compared to most individual models.

#### Future changes in annual mean precipitation

Climate models project the continuation of human-induced climate change in the twenty-first century and beyond. The bias-corrected and statistically downscaled NEX-GDDP models are often of greater relevance for impact, adaptation, and vulnerability applications to emphasize future climate projections. The MMM spatial patterns over the Arabian Peninsula and UAE area averages are reported for the historical and future periods.

**Figure 7:**
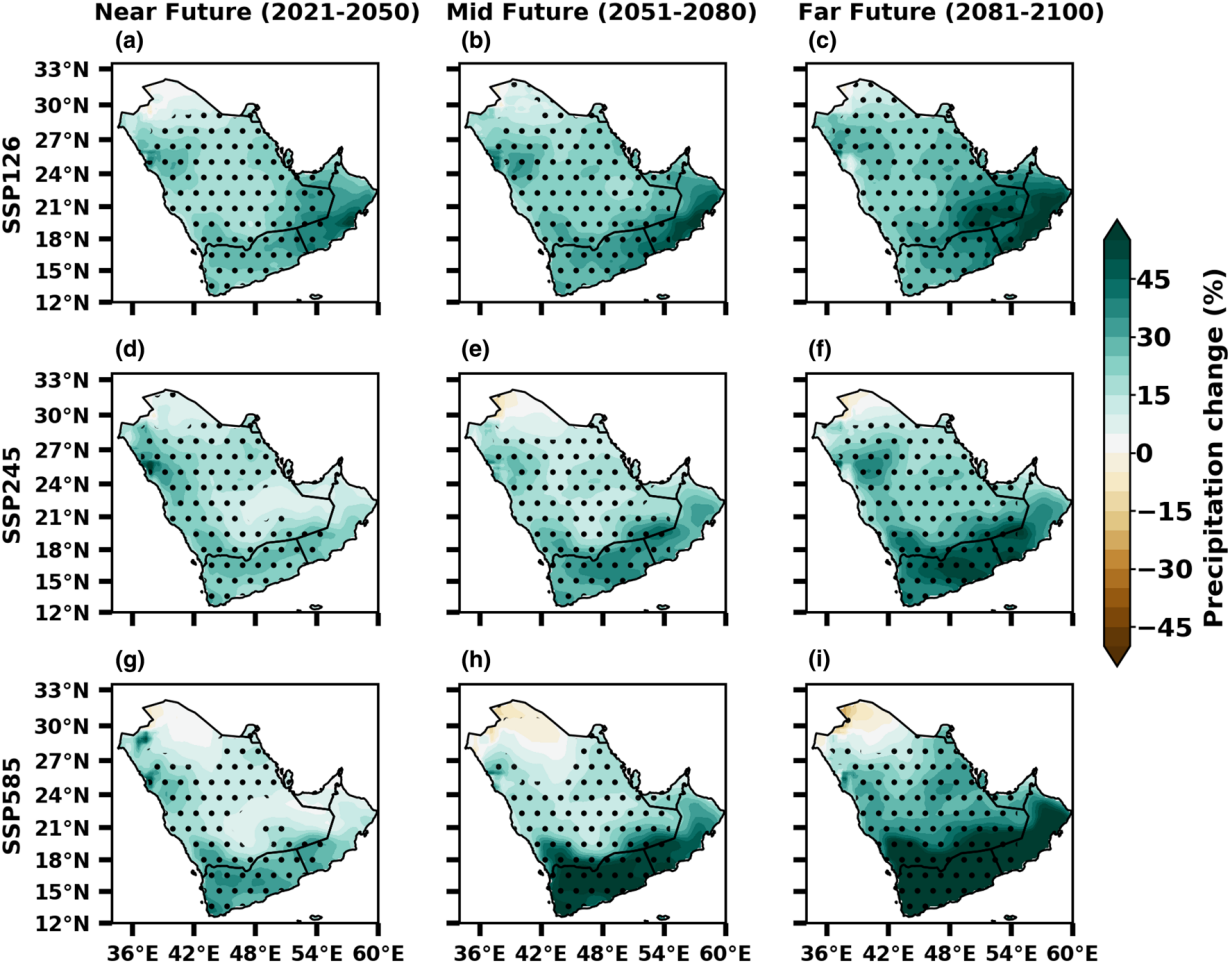
Future changes in annual total precipitation (%) from SSP1-2.6 (low emission), SSP2-4.5 (medium emission) and SSP5-8.5 (high mission) scenarios of CMIP6 during near, mid and far future with reference to the baseline 1985–2014. The stippling (dotted) regions represent the changes are statistically significant at 0.05 level.

Figure 7 depicts precipitation changes under three SSP scenarios of near, mid, and far future climates compared to the historical period. Results indicate that all the SSP scenarios show a significant increase in precipitation over most of the peninsular regions in the future, particularly intense from the mid-future onwards to the end of the twenty-first century. The Arabian Peninsula display a strong contrast in projected precipitation changes, with a significant increase in the southern and southeastern parts (Fig. 7). Drying is also projected but somewhat less in the NW parts, which agrees with previous studies on this region^67,75–79^. In addition, a slight increase in total precipitation may significantly increase relative (percentage) changes. On average, the precipitation is expected to increase by 20–40% towards the end of the century, and the projected signals are enhanced in the SSP5-8.5 scenario. Though the magnitude of actual rainfall increase may be small due to low rainfall received in the region, this projected surge in average annual precipitation can result in augmented occurrence and intensity of rainfall leading to flash floods and inundation. A study by AGEDI mentioned that rainfall is projected to increase over much of UAE and the Hajar mountains^80^. The results show a significant increase in precipitation, with a substantial magnitude of more than 40% expected in the southeastern parts. Overall, the projected increase in precipitation is consistent with the CMIP5 & 6 projections over the Arabian Peninsula, as shown in^67,74,76^.

**Figure 8:**
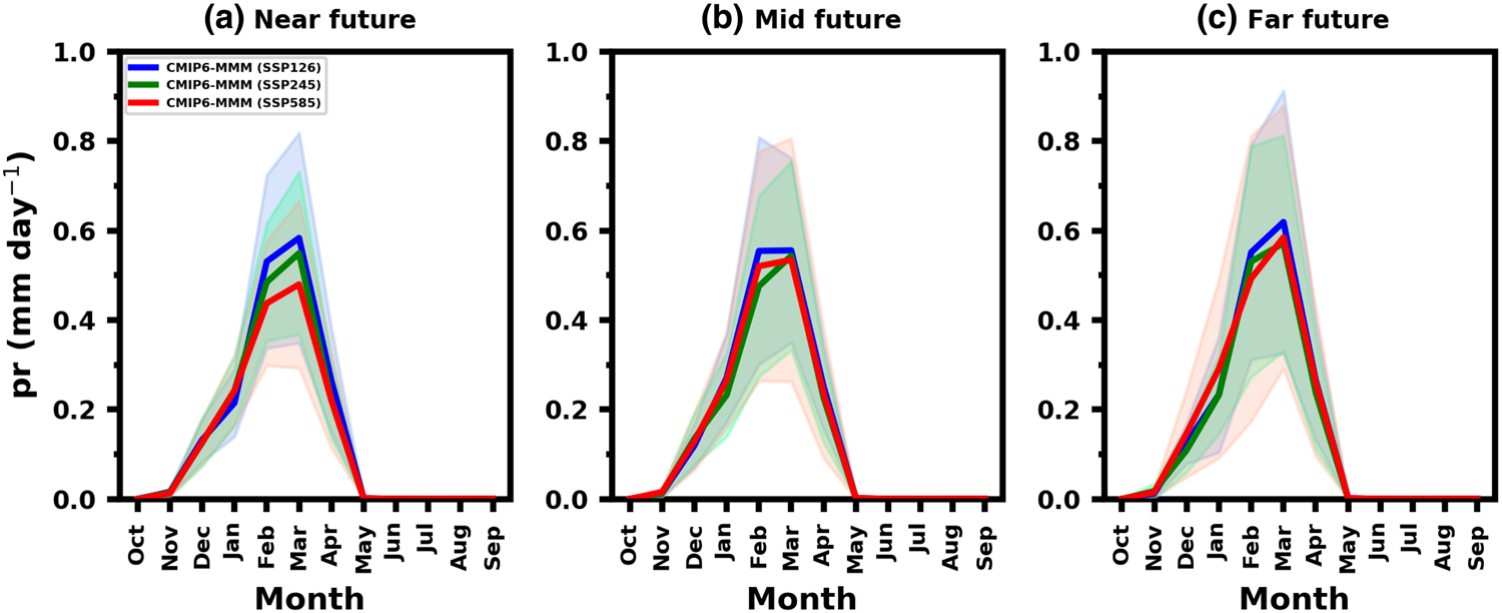
Area averaged UAE mean monthly future precipitation change relative to baseline period (1985–2014). The average rainfall (mm/day; solid lines) and the associated uncertainty range (shading) for near (2021–2050), mid (2051–2080) and far (2081–2100) future under SSP1-2.6, SSP2-4.5 and SSP5-8.5 scenarios.

The MMM estimate of projected monthly precipitation changes over UAE indicates a slight increase during the winter months in the future time slices (Fig. 8). Under the SSP5-8.5 scenario, the long-term monthly change shows a distinct annual cycle towards the end of the century. While the MMM suggests that UAE precipitation is projected to increase during the winter months by the end of the century, it is important to note that the associated uncertainty range for the monthly changes in individual models is also notably high.

**Figure 9:**
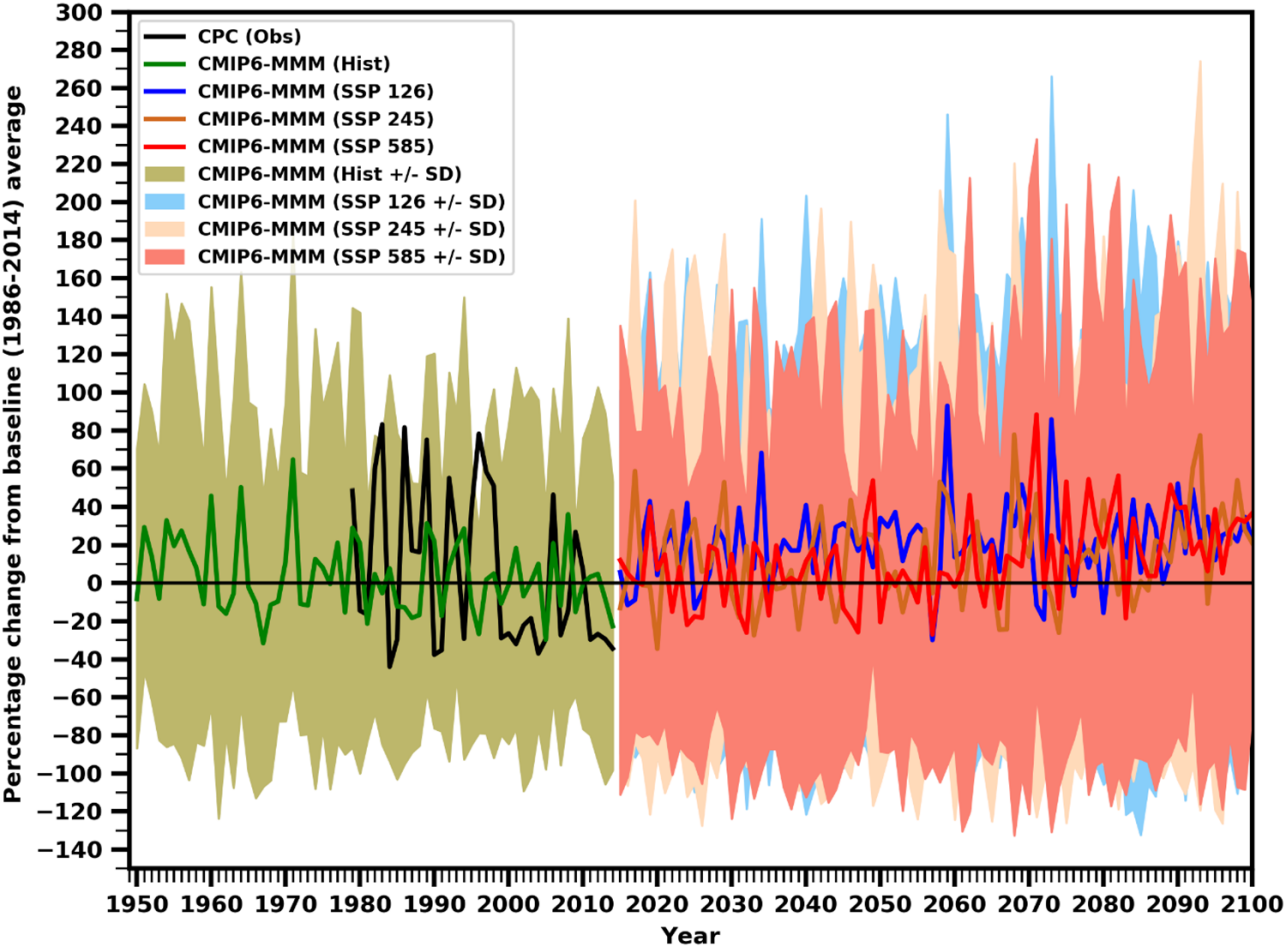
Time series of area averaged UAE annual total precipitation (%) anomalies (relative to 1985–2014) from CMIP6 models. The solid thick lines represent MMM and the shaded regions denote ± 1 SD from thirty models for historical, SSP1-2.6, SSP2-4.5 and SSP5-8.5 scenarios.

Projected annual precipitation changes (%) along with uncertainty in the model spread based on the mean ± S.D range for the three scenarios over UAE are presented in Fig. 9. Regionally, precipitation has been markedly affected by global warming, characterized by far more complicated responses than temperature. The historical MMM simulations well captured the observed interannual variability. However, there are significant model-to-model differences in the magnitude. The downscaled projections consistently depict interannual variations in the future across all the SSP scenarios, but they do not exhibit a consistent trend. The interannual variability and spread among the models in terms of precipitation changes are more pronounced, indicating a significant level of uncertainty in the projected precipitation patterns. Of utmost significance, the uncertainties associated with these projections diminish substantially at the regional scale. On average, the projected mean annual precipitation over most of the UAE is expected to increase by about 15 -30% during the current century under all the SSPs compared to the historical period, indicating the annual total precipitation of this region likely to increase significantly (Fig. 9). Overall, a slight wetting tendency may happen in individual years. These wetter than the present-day mean conditions result from the increased variability, combined with alterations in atmospheric thermodynamics and circulation drivers. The UNDP report projected that rainfall would be 10–25% by 2050, and it may increase by 22–45% towards the end of the twenty-first century relative to the baseline period (1961–1990)^81^. Results suggested that precipitation would increase in the future under global warming. In particular, Douville et al.^82^ and Seneviratne et al.^83^ stated that precipitation extremes would increase in most of the regions across the globe (high confidence), even where seasonal mean precipitation is projected to decrease (medium confidence). Numerous prior studies, including those outlined in the Intergovernmental Panel on Climate Change (IPCC) report^1^, have indicated that the northward movement of the Intertropical Convergence Zone (ITCZ) in the future could contribute to the observed increase in precipitation over the southern regions of the Middle East and North Africa (MENA) area^84^.

The annual total precipitation (area averaged over UAE) in the historical period is around 77 mm, increasing slightly above this historical estimate in future periods. The MMM for the near future indicates that the annual rainfall in the UAE may vary between 79 ± 15 mm and 91 ± 13 mm, respectively. In the mid-future, it ranges from 93 ± 13 mm to 92 ± 17 mm, and in the far future, it was between 92 ± 14 mm and 96 ± 17 mm, respectively. Table 3 provides a detailed insight into these changes with associated likely ranges under different SSP scenarios for the near, mid, and far future relative to the base period.

**Table 3:** The MMM estimate of projected annual precipitation (mm) over UAE and the associated associate standard deviation. The values in parenthesis show the standard deviation.

Scenario	Annual mean precipitation (mm)		
	Near future	Mid future	Far future
SSP1-2.6	91 (11)	93 (13)	92 (14)
SSP2-4.5	84 (14)	86 (16)	89 (17)
SSP5-8.5	79 (15)	92 (17)	96 (17)

The projected precipitation indicate a net increase in the future compared to the simulated precipitation during the historical period. The MMM projects a range of increased precipitation by the end of the twenty-first century, varying from 15 to 35% for the different SSP scenarios.

#### Future changes in extreme precipitation indices

This study also considered pertinent precipitation extreme indices helpful in assessing the societal implications of potentially significant changes caused by the destructive effects of extreme events in response to global warming. It is crucial to investigate the spatial and temporal patterns of projected changes in the near future, extending until the end of the century, to adequately plan for mitigation measures.

**Figure 10:**
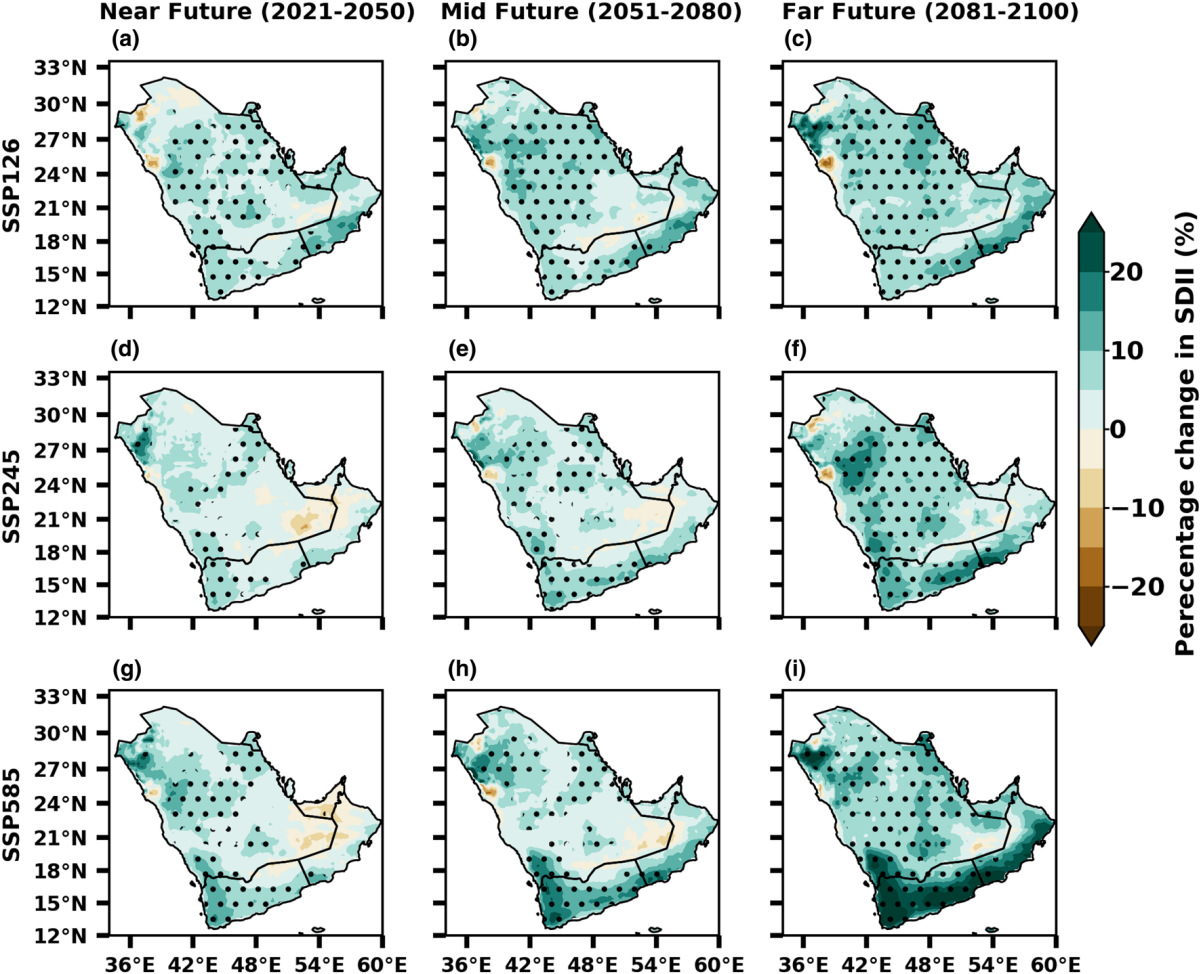
Future changes in precipitation intensity (%) from SSP1-2.6 (low emission), SSP2-4.5 (medium emission) and SSP5-8.5 (high mission) scenarios of CMIP6 during near, mid and far future with reference to the baseline 1985–2014. The stippling (dotted) regions represent the changes are statistically significant at 0.05 level.

The MMM shows an increase in intensity (SDII) under the SSP5-8.5 scenario compared to the SSP1-2.6 and SSP2-4.5 scenarios, particularly from mid-future onwards (Fig. 10), far future is more prominent with intense extreme events than in the near and mid-future periods. Notably, significant differences are projected for the increase in intensity (SDII) over the Arabian Peninsula in all the scenarios. The projected change in intensity is expected around − 5 to 25% compared to the baseline in all scenarios. In all scenarios, the projection in the near future climate demonstrates less intensity in the UAE region. This rise in intensity is reliable with the Clausius–Clapeyron relationship, which implies that the overall moisture content in the atmosphere plays a significant role in driving changes in the intensity of extreme precipitation events. Therefore, it is expected to change at a rate of ∼ 6 to 7% per degree of warming^5,85^. However, when it does begin to rain, the added moisture results in more extreme precipitation events^86^. According to recent study conducted by Bador and Alexander^87^, it has been suggested that both the annual precipitation totals and extreme precipitation events are expected to intensify across most land regions worldwide by the end of the twenty-first century. These changes are attributed to the rise in anthropogenic emissions and the resulting global warming.

**Figure 11:**
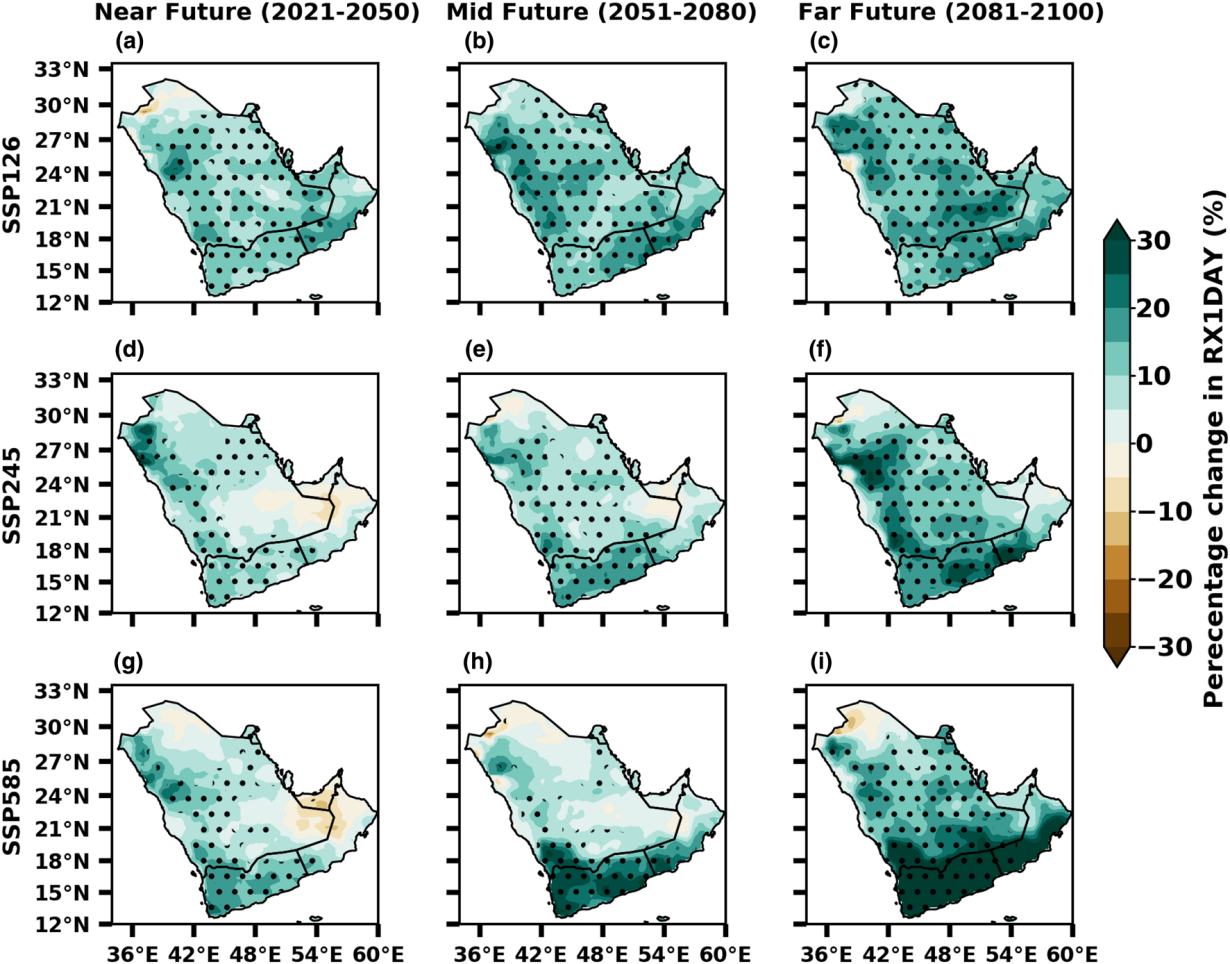
Future changes in 1-day highest precipitation (%) from SSP1-2.6 (low emission), SSP2-4.5 (medium emission) and SSP5-8.5 (high mission) scenarios of CMIP6 during near, mid and far future with reference to the baseline 1985–2014. The stippling (dotted) regions represent the changes are statistically significant at 0.05 level.

The ONE-day highest precipitation amount (RX1DAY) is anticipated to rise in the Arabian Peninsula (Fig. 11), indicating an increased chance of showers on a smaller scale towards the end of the period. In addition, the possibility of floods may upsurge during the far future (2081–2100) compared to the near future (2021–2050) due to the increase in RX1DAY precipitation. From a geographical perspective, the RX1DAY index, which represents the maximum daily precipitation, is primarily projected to increase in the southern and southeastern regions of the Arabian Peninsula compared to other areas. Conversely, a slight increase in RX1DAY is expected in the northeast and certain eastern parts under all three SSP scenarios. Overall, the spatial distribution of RX1DAY indicates that precipitation will become more sporadic and intense in the future. Moreover, the projected changes in RX1DAY, as indicated by the Multi-Model Mean (MMM), exhibit a notable variation in spatial distribution across the Arabian Peninsula. The highest response of increasing RX1DAY is due to global warming, which was noticed in regions of low latitudes (e.g., south and southeast).

**Figure 12:**
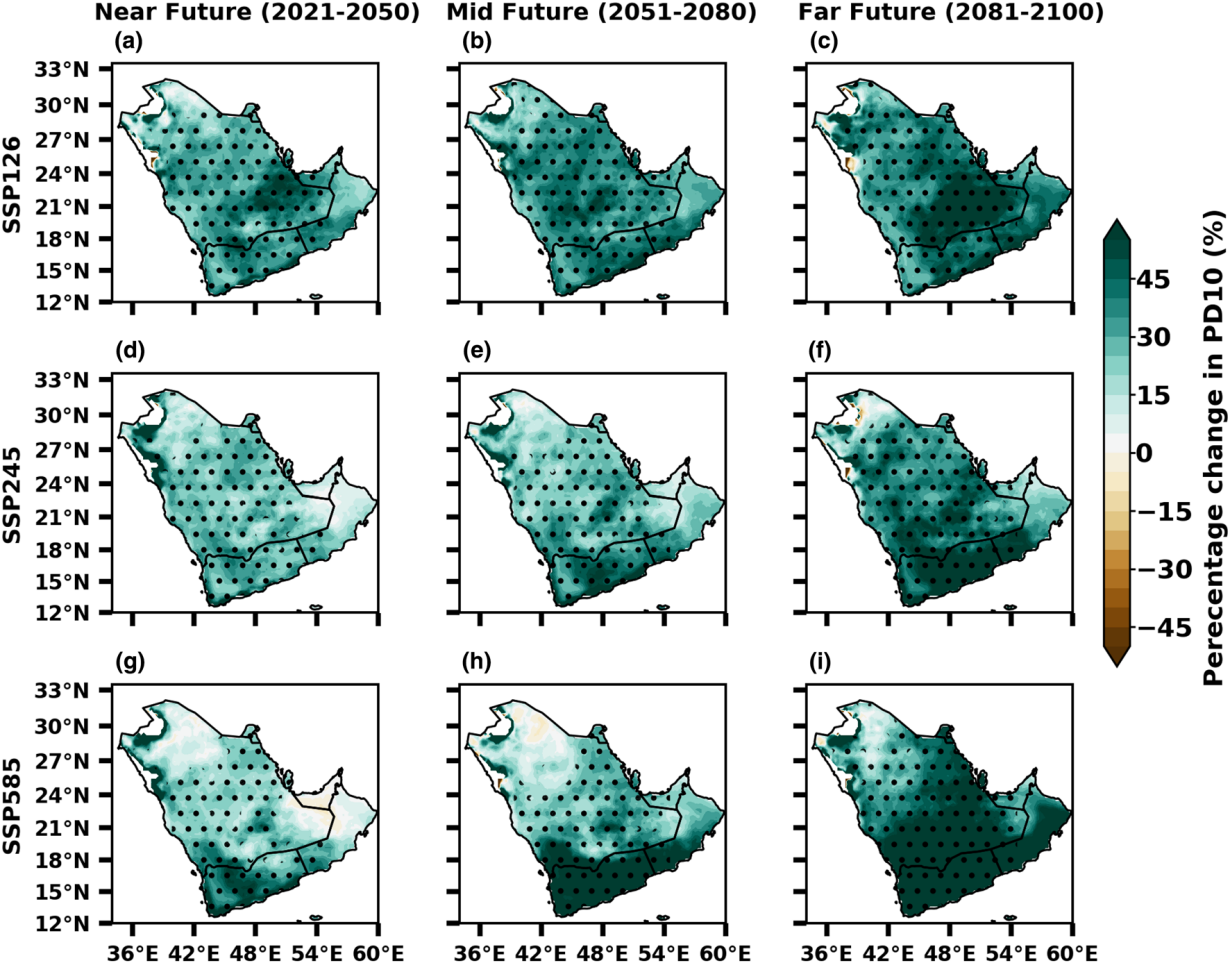
Future changes in more than 10 mm precipitation days (%) from SSP1-2.6 (low emission), SSP2-4.5 (medium emission) and SSP5-8.5 (high mission) scenarios of CMIP6 during near, mid and far future with reference to the baseline 1985–2014. The stippling (dotted) regions represent the changes are statistically significant at 0.05 level.

The occurrence of heavy precipitation days (≥ 10 mm) is likely to go upwards over most of the areas in the peninsula, mainly a noticeable increase in the southern and southeastern regions, indicating the rising frequency, which was clearly shown in all SSPs by MMM. There is a general increment in heavy precipitation days of around 10–50% from the mid to far future. Notably, in the SSP5-8.5 scenario, it is strengthened and covers most of the areas in the peninsula (Fig. 12). The findings of the study indicate that the Arabian Peninsula may witness an increase in extreme precipitation events. This suggests that there may be a significant increase in precipitation on wet days across most of the land area towards the end of the century. It is important to note that the projected increase in heavy precipitation days contributes to the overall increase in rainfall anticipated in the future.

**Figure 13:**
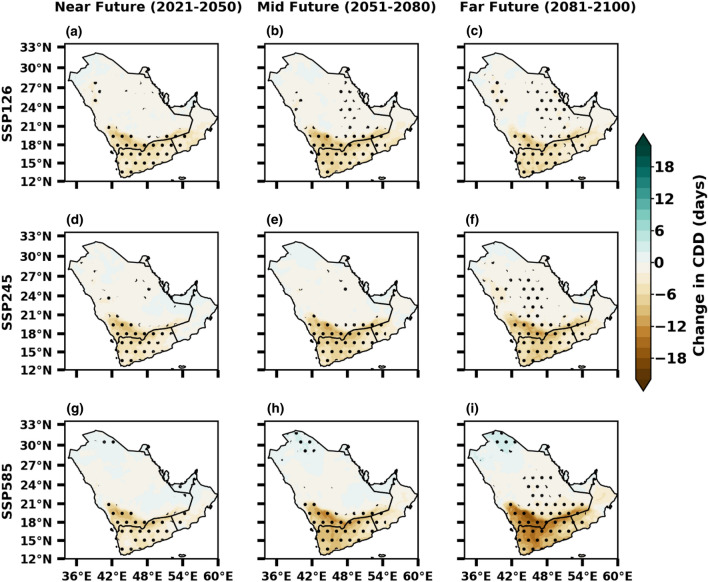
Future changes in dry days (days) from SSP1-2.6 (low emission), SSP2-4.5 (medium emission) and SSP5-8.5 (high mission) scenarios of CMIP6 during near, mid and far future with reference to the baseline 1985–2014. The stippling (dotted) regions represent the changes are statistically significant at 0.05 level.

Further, we considered the duration indices, such as CDD, which define the periods of dryness. The MMM of CDD depicts drying patterns over the peninsula reduced up to 20 days from near to far future compared with the baseline under three SSPS scenarios, which may be advantageous from an agricultural point of view (Fig. 13). The CDDs are projected to decrease over most of the peninsula due to the increased rainfall, implying that the rainy days may be more vigorous. Primarily, the CDDs are projected to decrease in most parts of the peninsula under SSP1-2.6, reduce in southern and southeastern, and increase in northern regions under SSP2-4.5. The distributions of precipitation changes projected under both SSP5-8.5 and SSP2-4.5 scenarios exhibit similar patterns. There is a noticeable decrease in precipitation over southern and southeastern areas, with the changes exceeding 10 days. The overall changing precipitation patterns across the Arabian Peninsula are similar among the three scenarios, although the magnitude of variation becomes larger with higher SSP scenarios. The changes in extreme precipitation events are more pronounced under the high-emission scenario compared to the other two scenarios. In general, most precipitation indices show an increasing trend over the Arabian Peninsula in the multi-model mean (MMM) projections for the twenty-first century across different SSP scenarios. These findings agree with Al Mozuri et al.^8^, who projected an increase in annual precipitation and extreme events over the broader peninsular region. The results from this study indicate that extreme precipitation will become more common in the future in Arabian Peninsula, consistent with the previous studies^1,88,89^.

**Figure 14:**
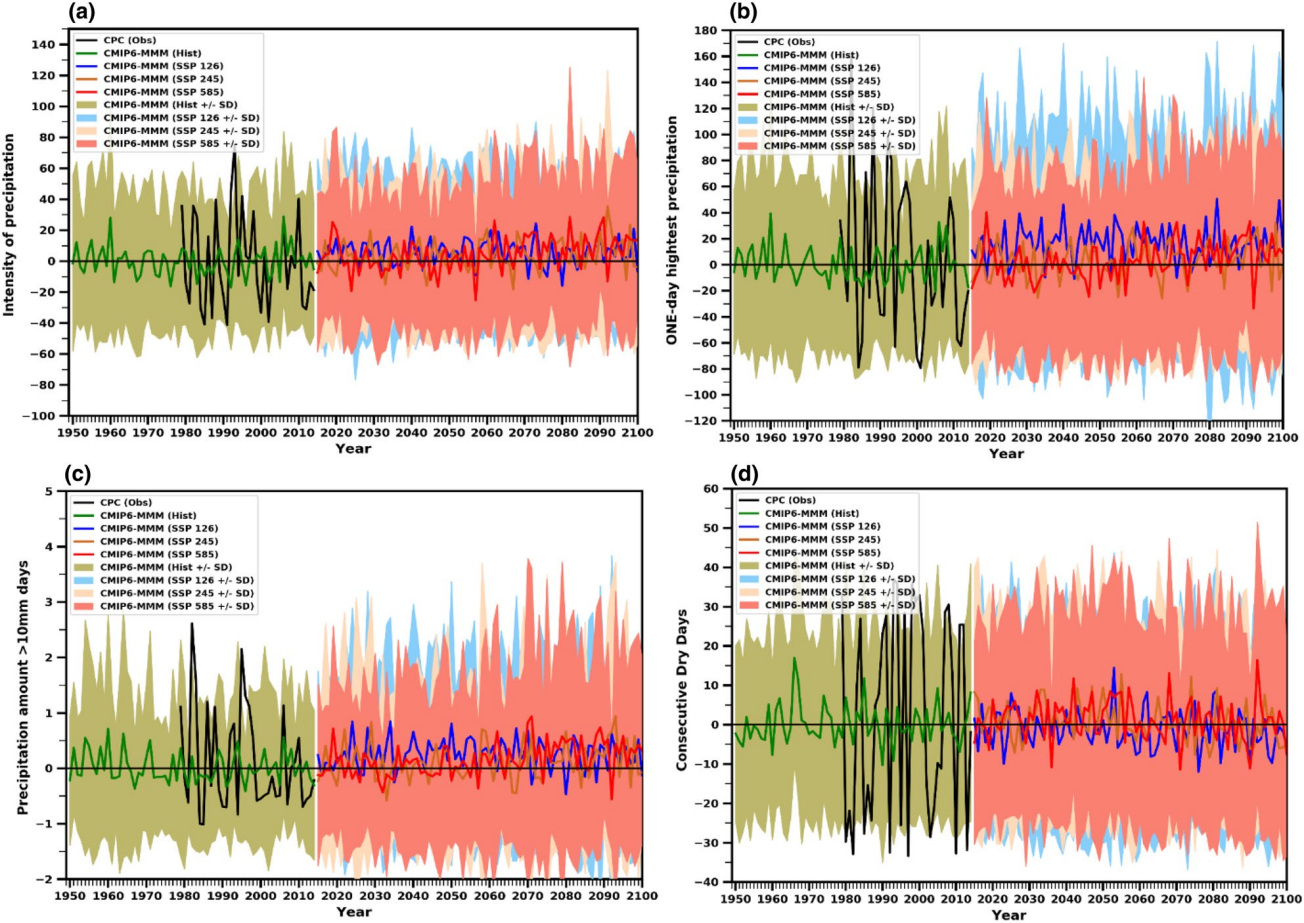
Time series of area averaged UAE annual (**a**) SDII, (**b**) RX1DAY, (**c**) PD10mm and (**d**) CDD anomalies (relative to 1985–2014) from CMIP6 models. The solid thick lines represent MMM and the shaded regions denote ± 1 SD from thirty models for historical, SSP1-2.6, SSP2-4.5 and SSP5-8.5 scenarios.

The Fig. 14 summarizes the time series in annual precipitation extremes over the UAE in the future years under SSP1-2.6, SSP2–4.5, and SSP5–8.5 scenarios relative to the historical period. The precipitation indices of SDII, RX1DAY, and PD10mm are projected to increase significantly under the three scenarios in the future (Fig. 14a–c). The duration indices, such as CDD, show a decreasing tendency (Fig. 14d). The projected increase in SDII, RX1DAY, and PD10mm is approximately 20 days, 10 mm, and 0.5 days, respectively, under the SSP5–8.5 scenario by the end of the century. However, CDD is expected to decline by approximately 5–15 days under a high-emission scenario. The increase and decrease patterns may occur in these indices, with depict large variability across the time series, with patterns of increase and decrease expected to occur. According to the findings of this study, the United Arab Emirates (UAE) is projected to experience changes in extremes, including a decrease in consecutive dry days (CDD) under the SSP5–8.5 scenario. The study suggests that regional atmospheric circulation changes and other large-scale dynamical factors can play a significant role in amplifying or inhibiting the intensification of extreme events in the region. These factors can influence the occurrence and characteristics of extreme weather events in UAE. In contrast, an upward trend in SDII, RX1DAY, and PD10mm is projected in the future. This increase in extreme precipitation events is a major threat to flood management and surface water control of large and critical watersheds, which will likely affect most sectors, such as agricultural productivity and societal infrastructure. Similarly, the study indicates that the persistence of wet periods, as represented by CDD, is expected to intensify under the SSP5–8.5 scenario. This projected increase in wet periods can positively influence agricultural production in the region. As agriculture in UAE heavily relies on rainfall and temperature, the anticipated changes in precipitation patterns, influenced by anthropogenic factors and internal variability, will likely contribute to increased agricultural productivity.

**Figure 15:**
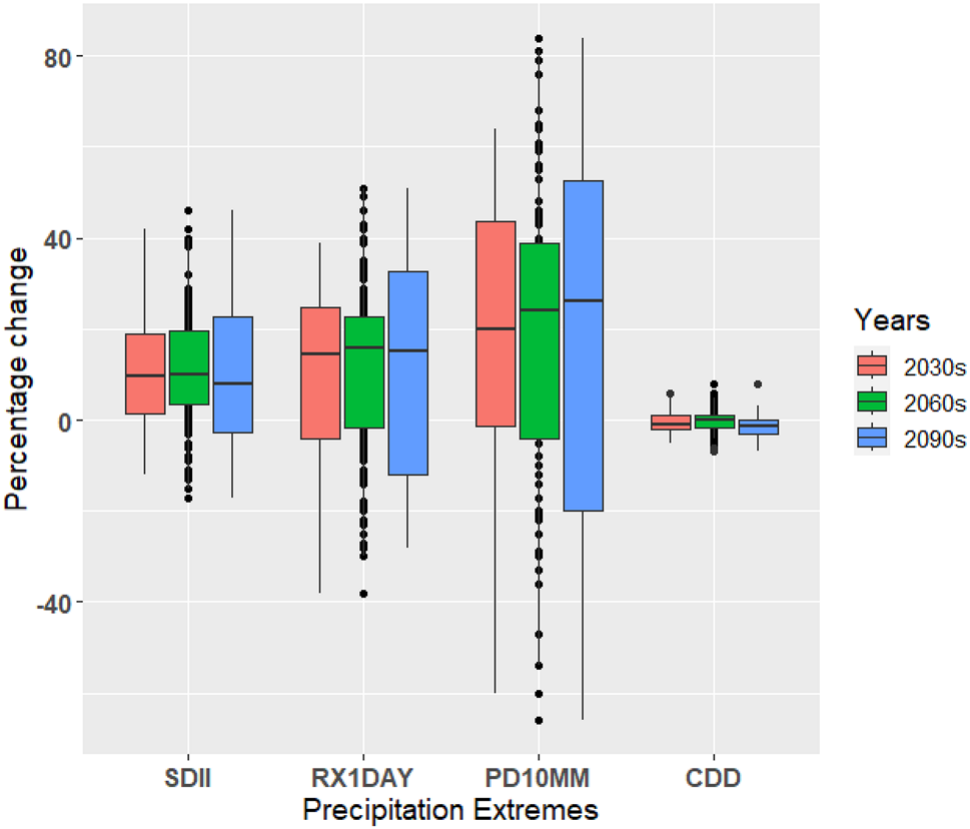
Boxplots of regionally averaged precipitation extreme indices over UAE for SSP1-2.6. Boxes indicate the interquartile model spread (25th and 75th quantiles), with the horizontal line indicating the multi model median and the whiskers showing the total intermodel range.

**Figure 16:**
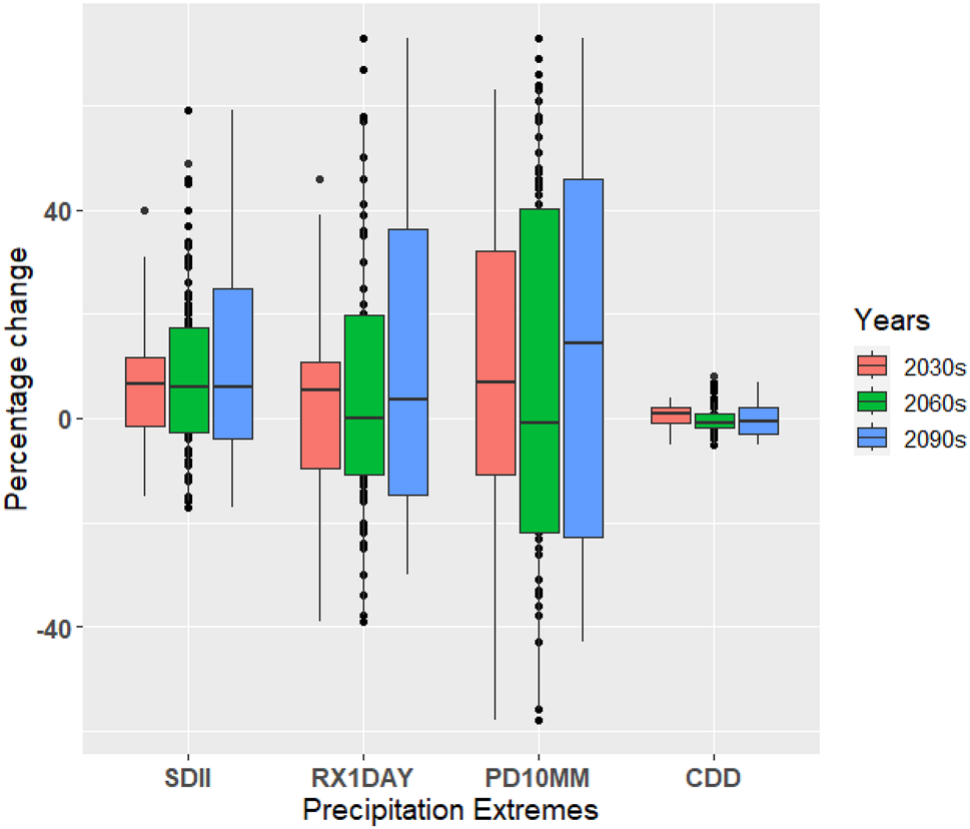
Boxplots of regionally averaged precipitation extreme indices over UAE for SSP2-4.5. Boxes indicate the interquartile model spread (25th and 75th quantiles), with the horizontal line indicating the multi model median and the whiskers showing the total intermodel range.

**Figure 17:**
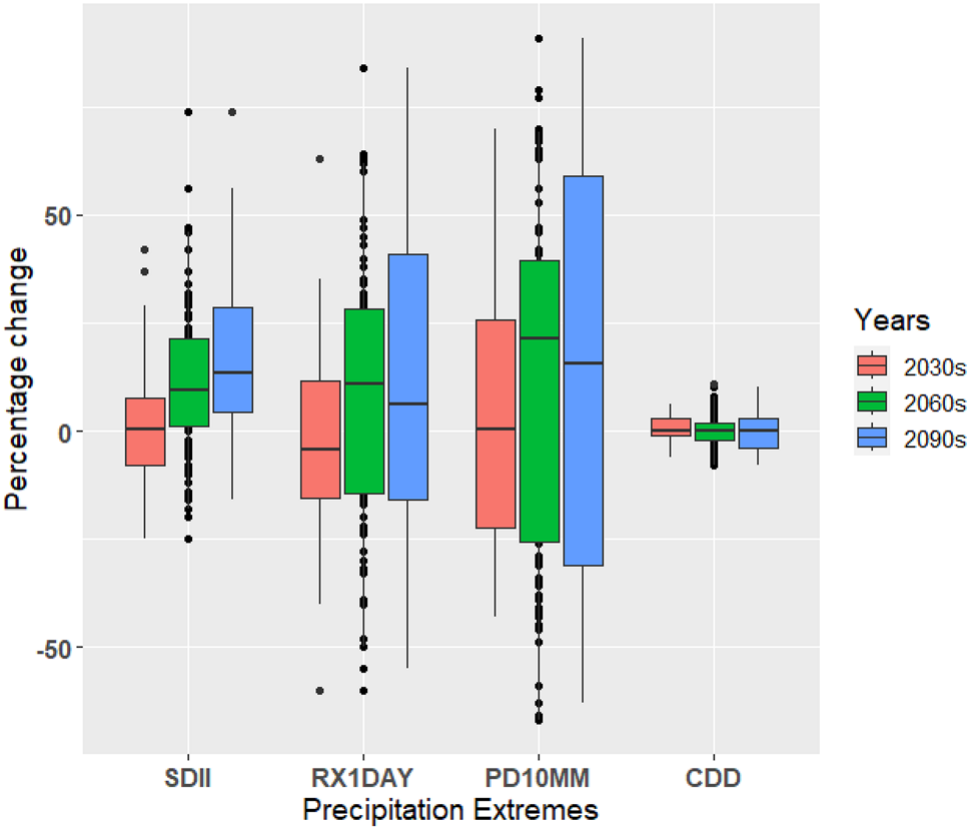
Boxplots of regionally averaged precipitation extreme indices over UAE for SSP5-8.5. Boxes indicate the interquartile model spread (25th and 75th quantiles), with the horizontal line indicating the multi model median and the whiskers showing the total intermodel range.

A thorough comprehension of precipitation changes is crucial for countries like the UAE, as an increase in extreme precipitation can have significant implications for the energy, tourism, and agriculture sectors. In this study, we examined the box-and-whisker plots depicting the percentage changes in precipitation intensity (SDII), maximum one-day precipitation (RX1day), precipitation amount exceeding 10 mm (PD10MM), and consecutive dry days (CDD) compared to the baseline period. These plots provide insights into the performance of climate models in simulating precipitation patterns and help assess the inherent uncertainties associated with these projections (Figs. 15, 16, and 17). Changes in the precipitation indices under a warming scenario relative to the baseline period indicate progressive wetting across the UAE, accompanied by increased heavy precipitation events and reduced dry spell events (Figs. 15, 16, and 17). The median changes in the number of SDII (precipitation intensity) are generally small in magnitude. Relative to the baseline period, median changes under three scenarios increase from near to far future periods (Figs. 15, 16, and 17). The increase in the magnitude of SDII is very significant, given the short-lived and convective nature of rain-bearing weather systems in the UAE. The combination of extreme precipitation events and potentially shorter heavy precipitation spells could have significant implications for various economic sectors, with the agricultural sector being particularly vulnerable. The average country ensemble median change in one-day maximum precipitation (RX1DAY) under three warming scenarios relative to the baseline indicates a general increase across UAE as the models progress to warmer climates. For RX1DAY, the changes are most significant between the three warming levels in UAE. Event-based precipitation indices, specifically PD10MM, exhibit more significant variability than other indices. This variability is more pronounced in the SSP2-4.5 scenario compared to the SSP5-8.5 scenario. Changes in the frequency of heavy (PD10MM) rainfall events show any substantial changes over UAE.

The CDD median decreases in all the periods for the SSP1-2.6, SSP2-4.5, and SSP5-8.5 scenarios, respectively, the largest of the changes. On average, UAE is projected to experience median decreases of around 5% of days in CDD under the respective warming scenarios. Across the ensemble members, there is widespread agreement regarding the direction of change in CDD. Over 80% of the members consistently show a decrease in CDD across the three warming periods, indicating a reduction in consecutive dry days throughout the country. The CDD decreased under the SSP5-8.5 compared to the SSP1-2.6 and SSP2-4.5 scenarios. During the mid-future (the 2060s), the CDD displays slight variability than in the near future (2030s) in the SSP2-4.5, whereas it continuously decreased in the SSP5-8.5 scenario. The change in the number of consecutive dry days shows a statistically significant decrease across all periods for all scenarios. The reductions in CDD imply wetting with increased precipitation, as noted by Siderius et al.^90^ and Sillmann et al.^54^. The discrepancy in consecutive dry days (CDD) indicates that the disparity in precipitation between the current climate and the emission scenarios is statistically significant. This can be attributed to the fact that even small percentage changes in total annual rainfall can significantly impact arid regions. The extreme indices, such as precipitation extremes, exhibit a noticeable increase with noticeable variations during different future periods. Bador et al.^91,92^ (2018, 2020) and Pfahl et al.^93^ presented a widespread intensification of total and extreme precipitation in the mid-to-high latitudes of the Northern Hemisphere that has been influenced by the thermodynamic response of the increasing surface temperatures. The contribution of the precipitation extremes supports the projected increase in the annual precipitation total for the study region^94–98^.

## Summary and conclusions

This study utilizes thirty global climate models datasets from the National Aeronautics and Space Administration (NASA) Earth Exchange Global Daily Downscaled Projections (NEX-GDDP). These data sets are statistically downscaled, bias-corrected, and provided at a high resolution of 0.25° × 0.25°.The changes are analyzed under SSP1-2.6, SSP2-4.5, and SSP5-8.5 scenarios. These datasets are derived from the new generation of state-of-the-art GCM simulations made under Coupled Model Intercomparison Project Phase 6 (CMIP6). This work is the first attempt to use NEX-GDDP models data to project regional precipitation regimes over Arabian Peninsula. This study performed a comprehensive analysis of projected changes in the precipitation regime in the near (2021–2050), mid (2051–2080), and far (2081–2100) future relative to the historical model simulations (1985–2014). For policy formulation, it is essential to study the behavior of precipitation changes in the present and future global warming levels over the UAE.

The observations show that there has been unequivocal warming over the Arabian Peninsula in the last four decades. The annual mean precipitation is of the order of 20 to 130 mm/year over the coastal and mountain regions of the peninsula during 1979–2022. In most places in the peninsula, precipitation shows a decreasing tendency. The extreme precipitation events do not show any significant trend (slight increasing/decreasing tendencies) over the entire UAE from 1979 to 2022. Overall, NEX-GDDP multimodal mean reasonably reproduces the mean climatological state of precipitation. The spatial distribution of changes in precipitation and associated extremes shows a significant increase over most of the Arabian Peninsula in the future, particularly intense from the mid-future onwards to the end of the twenty-first century under three SSP scenarios.

A significant increase in the annual mean precipitation is expected over most of the UAE by up to 30% during the current century under all the SSPs. Specifically, the spatiotemporal distribution of frequency and intensity precipitation extremes such as SDII, ONE-day highest precipitation, and days with rainfall exceeding 10 mm increases by 20 days, 10 mm, and 0.5 days per year across the UAE, respectively, while the consecutive dry days may decrease by 15 days in response to increased atmospheric moisture content toward the end of the century. The results show that the changes in extreme precipitation may enhance under a warming scenario relative to the historical period, indicating progressive wetting across UAE, accompanied by increased heavy rainfall under the high emission scenarios. Particularly, ONE-day highest precipitation (RX1DAY) is expected to increase, indicating an increased chance of rainstorms on a smaller scale over the region towards the end of the century. Changes in the precipitation indices under a warming scenario relative to the baseline period indicate progressive wetting across UAE, accompanied by increased heavy precipitation events and reduced dry spell events, particularly under the high emission scenarios. The variation in climate outcomes projected from different climate models and scenarios underscores the importance of our decisions and actions today in shaping UAE’s climate future.

A high-resolution dataset is essential to improve the insights into changes in the precipitation regime. The overall assessment highlights the importance of conducting more studies that are comprehensive and gathering information at regional and national levels, particularly in climate-sensitive regions. These regions are influenced by local factors such as topography, geographical location, and proximity to seas and oceans, which can significantly impact their climate. It is crucial to understand these local drivers to obtain a more accurate and detailed understanding of climate dynamics and potential climate change impacts in these areas. A firm grasp of historical climate trends and climate change projections at the regional scale is crucial in this endeavor, as many decisions, planning processes, and implementation efforts occur at the regional level. This current analysis will be helpful for impact studies and, ultimately, in devising future policies for adaptation and mitigation strategies in the region.

## Data Availability

This study used gridded daily precipitation data from NASA Earth Exchange Global Daily Downscaled Projections (NEX-GDDP), derived from the new generation of state-of-the-art GCM simulations made under Coupled Model Intercomparison Project Phase 6 (CMIP6). These NEX GDDP data sets are available at https://www.nccs.nasa.gov/services/data-collections/land-based-products/nex-gddp-cmip6. Observed daily precipitation data were taken from the Climate Prediction Center (CPC) developed by the National Oceanic and Atmospheric Administration (NOAA), available at https://psl.noaa.gov/data/gridded/. The datasets used and analyzed during the current study are available from the corresponding author upon reasonable request.
